# Bovine Papillomavirus Type 2 Infection and a Series of Mesenchymal Tumors of the Urinary Bladder in Cattle

**DOI:** 10.1155/2013/814635

**Published:** 2013-06-05

**Authors:** Manuela Martano, Franco Roperto, Rita de Cassia Stocco, Valeria Russo, Giuseppe Borzacchiello, Orlando Paciello, Valentina Iovane, Leonardo Leonardi, Paola Maiolino, Brunella Restucci, Serenella Papparella, Sante Roperto

**Affiliations:** ^1^Department of Veterinary Medicine and Animal Productions, Naples University Federico II, Via F. Delpino, 80137 Naples, Italy; ^2^Department of Biology, Naples University Federico II, Via Mezzocannone 16, 80134 Naples, Italy; ^3^Instituto Butantan, Laboratório de Genética, Avenida Vital Brazil 1500, 05503-900 São Paulo, Brazil; ^4^Department of Biopathological Sciences and Hygiene of Animal and Alimentary Productions, University of Perugia, Faculty of Veterinary Medicine, Via San Costanza, 4-06126 Perugia, Italy

## Abstract

This report describes the histopathology of two hundred and fifty-three mesenchymal tumors of the urinary bladder in cattle grazing on lands rich in bracken fern. Approximately 80% were hemangiomas and angiosarcomas. Hemangioma (capillary, cavernous, and large vessels) was the most frequent mesenchymal tumor and was more common than angiosarcoma. Although the appearance of endothelial cells can vary remarkably, epithelioid angiosarcomas, often containing multinucleated cells, were the most frequent malignant vascular tumors. Hemangiopericytoma and tumors of muscle and soft connective tissue origin, alone and/or in association with tumor-like lesions, were less frequently seen. Furthermore, forty-five cases of intravascular papillary endothelial hyperplasia (IPEH), a lesion not previously reported in the urinary bladder of cattle, were also described. Bovine papillomavirus type-2 DNA was amplified in tumor samples. Forty vascular tumors were investigated by dual-labeling immunofluorescence, and, for the first time, a coexpression of E5 and platelet-derived growth factor **β** receptor (PDGF**β**R) was shown to occur. The results show that the BPV-2 E5 oncoprotein binds to the activated form of the PDGF**β** receptor thus playing an important role in mesenchymal as well as epithelial carcinogenesis of the urinary bladder. Furthermore, these findings demonstrate that BPV-2 infects both epithelial and mesenchymal cells.

## 1. Introduction 

 Tumors of the urinary bladder are quite rare in cattle, representing approximately 0.01% of all malignant tumors [1]. However, their incidence increases greatly (>90%) in adult cattle, reared in geographic regions rich in bracken fern (*Pteridium* spp.) [2–4]. Prolonged ingestion of bracken fern produces a clinical syndrome known as chronic enzootic hematuria (CEH), being characterized by hematuria and anemia. In over 90% of cases, hematuria originates from tumors of the urinary bladder [1, 5]. It is well known that this plant plays an important role in causing bladder tumors in cattle, either directly and/or indirectly. Toxic principles of bracken fern are known to be immunosuppressive, mutagenic, clastogenic, and carcinogenic [6, 7]; the most important carcinogen appears to be a sesquiterpenoid named ptaquiloside (PT) [8]. It has been suggested that PT targets both the stroma and the urothelium of urinary bladder [9]. PT is responsible for alkylation of the codon 61 of H-ras gene thus leading to an uncontrolled urothelial cell proliferation and urothelial cell dysplasia [9–11].

 A strong synergism between bovine papillomavirus type 2 (BPV-2) and bracken fern has been shown in experimental bladder carcinogenesis [12], as well as in naturally occurring bovine bladder tumors [2, 4, 13, 14]. It is well known that PT can cause toxicity to the immune system, which could contribute to the activation of a latent and/or subclinical urothelial BPV-2 infection resulting in neoplasia [13]. PT is a powerful immune suppressor that is genotoxic for lymphocytes [11, 15–17] and depresses NK cell activities [18]. 

 Papillomavirus-associated bladder tumors are mainly urothelial in origin [4, 5, 19]. Numerous molecular pathways by which BPVs are able to transform urothelial cells are known to occur *in vivo*. It has been shown that BPV-2 is responsible for neoplastic transformation of urothelial cells via the activation of platelet-derived growth factor *β* receptor (PDGF*β*R) and/or of a tissue-specific protein named Calpain 3 [20, 21]. Mixed tumors (urothelial and mesenchymal) are frequently found in cattle grazing on fern-infested lands. In a recent survey, 331 out of 870 urinary bladder tumors (38%) showed a mesenchymal histogenesis and were found in cattle suffering from bovine enzootic hematuria [5]. Viral oncoproteins were found to be expressed also in vascular tumors of the bovine urinary bladder [22]. 

 The purpose of this paper was to report some histologic features of the mesenchymal neoplastic lesions of the urinary bladder of cattle naturally exposed to bracken fern and infected with BPV-2.

## 2. Material and Methods

### 2.1. Tumor Samples

 From 1999 to 2012, six hundred and fifty tumor samples of the urinary bladder were collected at public slaughterhouses from 4- to 30-year-old cattle that had suffered from chronic enzootic hematuria for several years. All the animals were known to graze on hilly/mountain pasturelands rich in bracken fern. For morphological investigations, the samples were fixed in 10% neutral buffered formalin and embedded in paraffin wax. Two hundred and fifty-three mesenchymal tumors were diagnosed on 4 *μ*m-ticked sections that were stained with hematoxylin and eosin. The neoplastic lesions were classified according to criteria given in the 2004 World Health Organization (WHO) Blue Book on the pathology and genetics of tumors of the human urinary system as well as in more recent surveys about the soft tissue tumors of the urinary bladder [23–26]. 

### 2.2. Immunofluorescence and Confocal Laser-Scanning Microscopy

Two-color immunofluorescence staining was performed on forty vascular tumors (hemangiomas), to assess the expression of E5 and PDGF *β* receptors. 

The sections were deparaffinized, rinsed in PBS, and heated in a microwave oven in citrate buffer to allow antigen unmasking. Slides were then preincubated with normal donkey serum diluted at 1 : 20 in PBS for 30 min and overlaid with polyclonal sheep anti-BPV-2 E5 diluted at 1 : 25 in PBS at 4°C, overnight, in a humid chamber. Then, a polyclonal goat anti-p-PDGF*β* receptor antibody (Santa Cruz Biotechnology) diluted at 1 : 25 in PBS was applied overnight. A secondary antibody Alexa Fluor 488 donkey anti-sheep (green) (Invitrogen, Molecular Probes) and a secondary antibody Alexa Fluor 546 donkey anti-goat (red) (Invitrogen, Molecular Probes), diluted at 1 : 50 in PBS, were applied for 2 h at room temperature. The slides were washed three times with PBS and mounted under aqueous medium (Sigma-Aldrich). For observation and photography, a laser-scanning confocal microscope (LSM-510; Zeiss) was used.

## 3. Results 

Two hundred and fifty-three out of six hundred and fifty bladder tumors were mesenchymal tumors (40%). Frequently, several different patterns of mesenchymal neoplasms coexisted. The size and location of neoplastic lesions varied greatly. In a few cases neoplasia was localized to a single site, but most tumors were shown to occur as multifocal lesions. Benign tumors (67%) were more common than malignant ones (33%). Mesenchymal tumors of the bladder are summarized in Table 1. 

### 3.1. Tumor-Like Lesions and Tumors of Vascular Origin

 Vascular tumors were seen in 204/253 cases accounting approximately for 81% of all bladder tumors. 

#### 3.1.1. Intravascular Papillary Endothelial Hyperplasia (IPEH)

**Figure 1:**
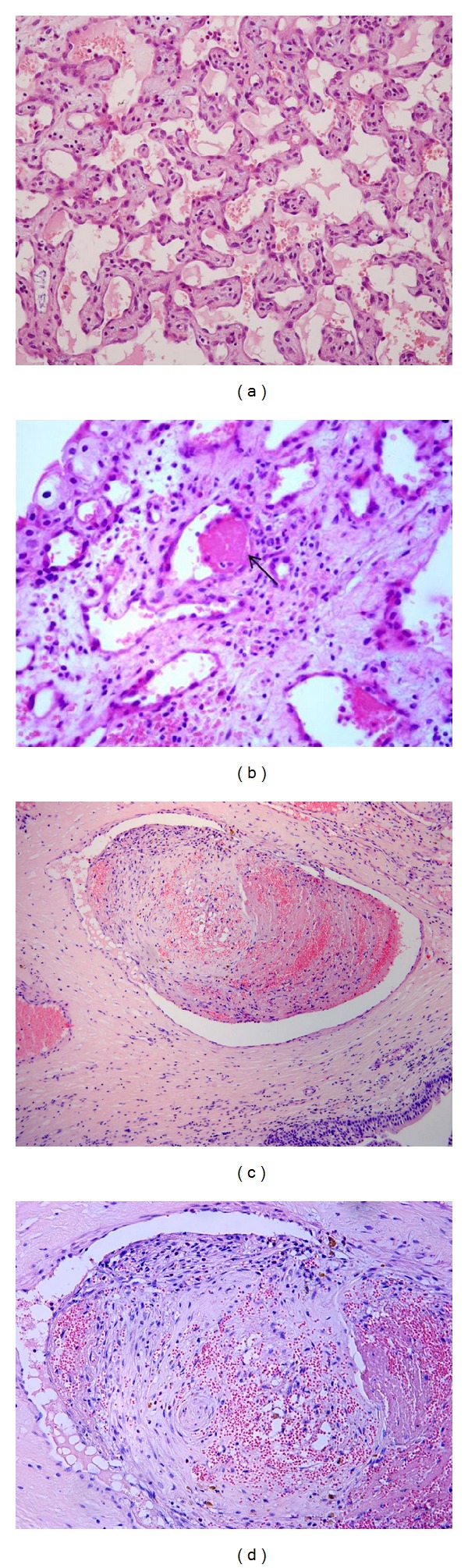
Intravascular papillary endothelial hyperplasia (IPEH). (a) Papillary structures within a vascular space, lined by monolayer and nonatypical endothelial cells, characterized this lesion (H&E, objective 20x). (b) Several intravascular fibrin deposits are seen (arrows) (H&E, objective 40x). (c) An exuberant, partly organized, and recanalyzed thrombus is shown (H&E, objective 10x). (d) As in (c) at higher magnification (H&E, objective 20x).

 First described by Masson as “vegetant intravascular hemangioendothelioma” [27], it is a benign, florid proliferation of endothelial cells [28]. Very likely it does not represent a true tumor; it is believed to be an exuberant organization and recanalization of a thrombus [29] and is a rare lesion of the bladder in humans [30]. To our knowledge, the only one case of IPEH in veterinary medicine has been reported in the conjunctiva of a horse [31]. In the urinary bladder of cattle, this lesion is commonly observed. Both the *de novo* “pure” form and, more frequently, the mixed type associated with benign and malignant vascular and urothelial tumors were seen. The exclusively intravascular nature of the process, the lack of atypical cells and aberrant mitoses, and the hyaline appearance of the papillae covered by monolayered cells are characteristic features of the lesions in cattle as well as in humans (Figure 1(a)). Intravascular deposits of fibrin were present (Figure 1(b)), and, in some cases, there were exuberant, partly organized, and recanalized thrombi (Figures 1(c) and 1(d)). 

#### 3.1.2. Hemangioma

**Figure 2:**
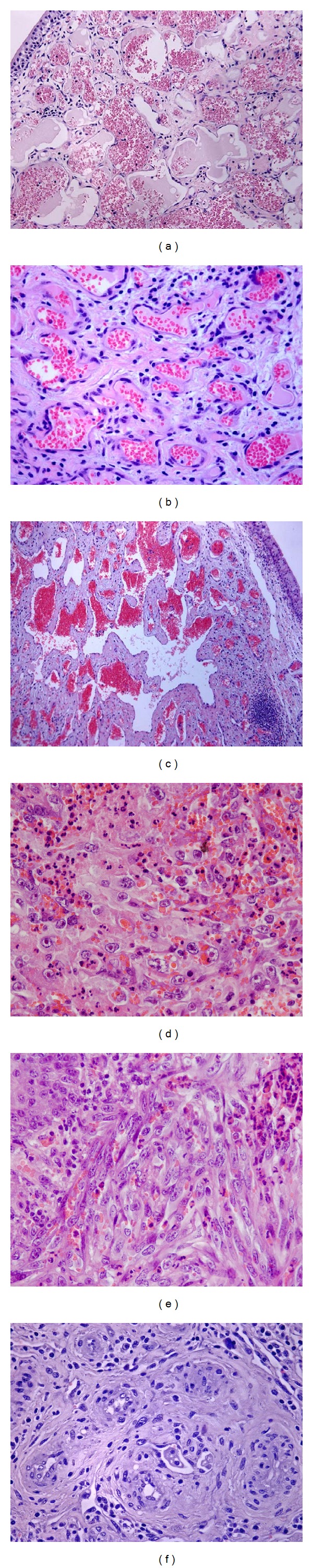
Tumors of vascular origin. (a) Cavernous haemangioma composed of vessels varying in size and shape (H&E, objective 20x). (b) Capillary hemangioma composed of small, rather uniform vessels (H&E, objective 20x). (c) Large-vessel hemangioma composed of intercommunicating vascular structures (H&E, objective 20x). (d) Epithelioid angiosarcoma characterized by round cells with vesicular nuclei with prominent nucleoli (H&E, objective 40x). (e) Kaposi-like hemangioendothelioma composed of spindle cells around vascular structures (H&E, objective 40x). (f) A particular staghorn appearance of the haemangiopericytoma (H&E, objective 40x).

 Hemangiomas in the present series were usually multiple and occurred anywhere in the bladder wall. The most common type of hemangioma was the *cavernous type*, which consisted of vascular lacunas, covered with endothelium, containing erythrocytes, and organized thrombi in the lumen (Figure 2(a)). Cavernous hemangioma has been observed alone or in association with the *capillary type*, consisting predominantly of closely packed aggregations of capillaries, usually of normal caliber, and separated by scant connective stroma (Figure 2(b)). Large-vessel hemangiomas were the less-common tumors and were generally composed of a combination of veins and arteries (*arteriovenous hemangiomas*) (Figure 2(c)).

 In humans, bladder hemangioma is a rare benign lesion and is generally considered as a congenital anomaly, not infrequently associated with the Klippel-Trenaunay or Sturge-Weber syndromes [32, 33]. Hemangiomas are rare in animals as well [1]. On the contrary, in cattle with bovine enzootic hematuria, this was the most common soft tissue neoplasm of the urinary bladder (143/253 cases, 57% of all mesenchymal tumors). As in humans [29], cattle hemangioma can be classified according to the caliber of vessels in capillary, cavernous, and large-vessel hemangiomas.

#### 3.1.3. Lymphangioma

 This tumor occurred rarely in our survey (2/253 cases, 0.8%) and consisted of bundles of plump smooth muscle cells proliferating in and around lymphatic channels.

#### 3.1.4. Angiosarcoma

 Angiosarcoma consists of anastomosing vascular channels lined by endothelial cells with marked atypia and prominent nucleoli. Few cases of angiosarcoma in the urinary bladder have been reported in humans. On the contrary, these tumors were identified in approximately 23% of the cattle in the present series (57/253 cases). Most of these tumors have an *epithelioid pattern* and were composed of cords or nests of endothelial cells with an epithelioid morphology that formed small vascular structures (Figure 2(d)). Aberrant and multinucleated cells were seen in some of them. However, despite the fact that they often appear to be composed of marked atypical cells, they rarely metastasize. We found only one angiosarcoma that had metastasized to distant organs. Recently four epithelioid hemangiosarcomas were described in the urinary bladder of cows with severe enzootic haematuria. Neoplastic cells, like endothelial cells, were immunohistochemically positive to factor-VIII-related antigen and ultrastructurally often contained cytoplasmic intermediate filaments, resembling epithelial cells [34]. 

#### 3.1.5. Hemangioendotelioma

 The morphology of this tumor appears to be in a category between hemangioma and hemangiosarcoma and could arise in several organ systems, skin, and soft tissue. There are few reported cases arising primarily in the bladder [35].

We reported two cases of hemangioendotheliomas (0.8%) of the urinary bladder of cattle suffering from chronic enzootic haematuria. They were classified as Kaposi-like hemangioendotheliomas since they were characterized by a proliferation of fusiform tumor cells with eosinophilic cytoplasm and elongated nuclei, showing moderate atypia, associated to features of capillary hemangioma (Figure 2(e)). Kaposi-like hemangioendothelioma is extremely rare, and it has recently been described in the urinary bladder in a cow [36].

#### 3.1.6. Hemangiopericytoma

 Histologically this neoplasm appears to be composed of polygonal or spindle-shaped cells packed around branching vascular channels, with staghorn configuration (Figure 2(f)). Nine cases (3.6%) were reported in the cattle of the present series (Table 1). A similar percentage has been reported by other authors [5]. Unlike cattle, hemangiopericytoma of the urinary bladder is exceptionally rare in man. A review of the literature revealed rare cases arising primarily in the urinary bladder [26].

#### 3.1.7. Glomus Tumor

Glomus tumor is composed of a proliferation of sharply demarcated modified smooth muscle cells, which are often arranged around dilated staghorn vessels. The cells contain a round nucleus and pale cytoplasm and generally low mitotic activity. The only case of multiple glomous tumor which has been reported in the present series was previously described by Roperto et al. [37].

### 3.2. Tumors of Muscle Origin

#### 3.2.1. Leiomyoma, Leiomyosarcoma, and Rhabdomyosarcoma

 In man, both benign and malignant tumors of smooth muscle origin are the most common mesenchymal tumors of the bladder [35]. Rhabdomyosarcoma of the bladder is relatively rare; however, the urinary bladder is believed to be the most common location for this type of tumor in the pediatric population since 90% of rhabdomyosarcomas in children are seen in the bladder [35]. Of the three types of rhabdomyosarcomas (embryonal, alveolar, and pleomorphic) by far the most common is the embryonal type [35, 38]. 

**Figure 3:**
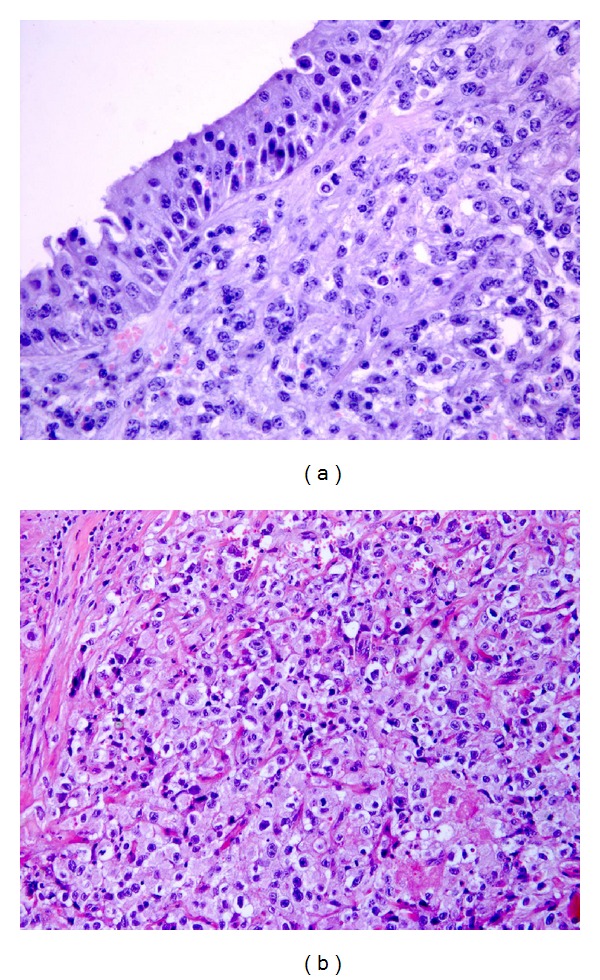
Rhabdomyosarcoma: (a) suburothelial (cambium layer) proliferation of round cells with scant cytoplasm, vesicular nuclei, and prominent nucleoli (H&E, objective 40x); (b) rhabdomyosarcoma characterized by small round and spindle cells, some of which showing a deeply acidophilic cytoplasm (H&E, objective 20x).

 Tumors of muscle origin represent approximately 6% (16/253) of the mesenchymal tumors of the present series (Table 1). We found five leiomyoma and eight malignant tumors, the microscopic patterns of which are consistent with the diagnosis of leiomyosarcoma. Histologically, these tumors are similar to those seen in other sites. We also found three cases of rhabdomyosarcoma. The microscopic patterns were characterized by a suburothelial (cambium layer) proliferation of round cells showing scant cytoplasm and vesicular nuclei containing prominent nucleoli. Rhabdomyosarcomas composed of small and spindle cells, some of them showing a deeply acidophilic cytoplasm, were also seen (Figures 3(a) and 3(b)).

### 3.3. Miscellaneous Mesenchymal Neoplastic Lesions of the Urinary Bladder

**Figure 4:**
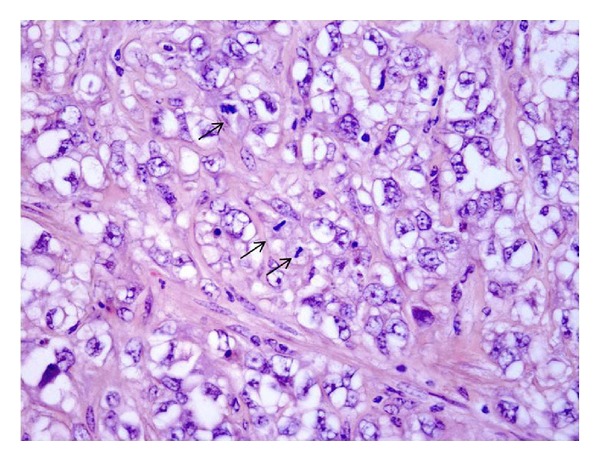
Bladder primary lymphoma composed of atypical cells showing vesicular nuclei, prominent nucleoli, and abnormal mitosis (arrows) (H&E, objective 40x).

We found other neoplastic lesions arising in the bovine bladder. Three cases of malignant lymphoma were composed of numerous pleomorphic, hyperchromatic and lymphocytic cells, embedded in a delicate edematous stroma (Figure 4). Two fibromas and three stromal fibrous reactions, two fibrosarcomas, six true myxomas, and seven stromal mixomatous reactions were also seen. 

**Figure 5:**
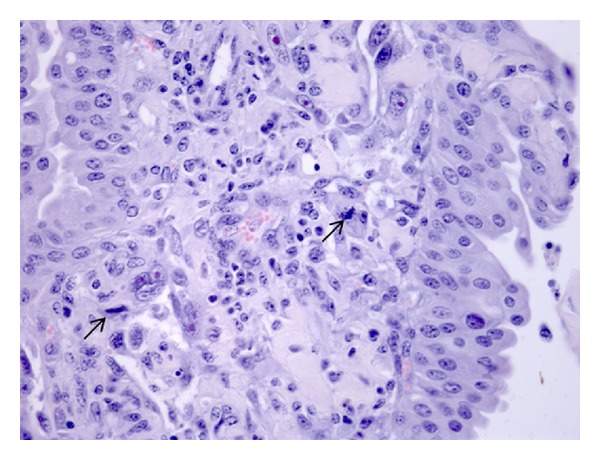
Carcinosarcoma shows an association of malignant epithelial and mesenchymal components. The latter is composed of large, abnormal cells with numerous mitoses (arrows) (H&E, objective 40x).

 Finally, three cases of carcinosarcomas, in which the mesenchymal component was composed of round cells with nuclear pleomorphism and hyperchromasia, were also documented (Figure 5).

### 3.4. Colocalization of E5 and PDGF b Receptors in Bladder Mesenchymal Tumours

**Figure 6:**
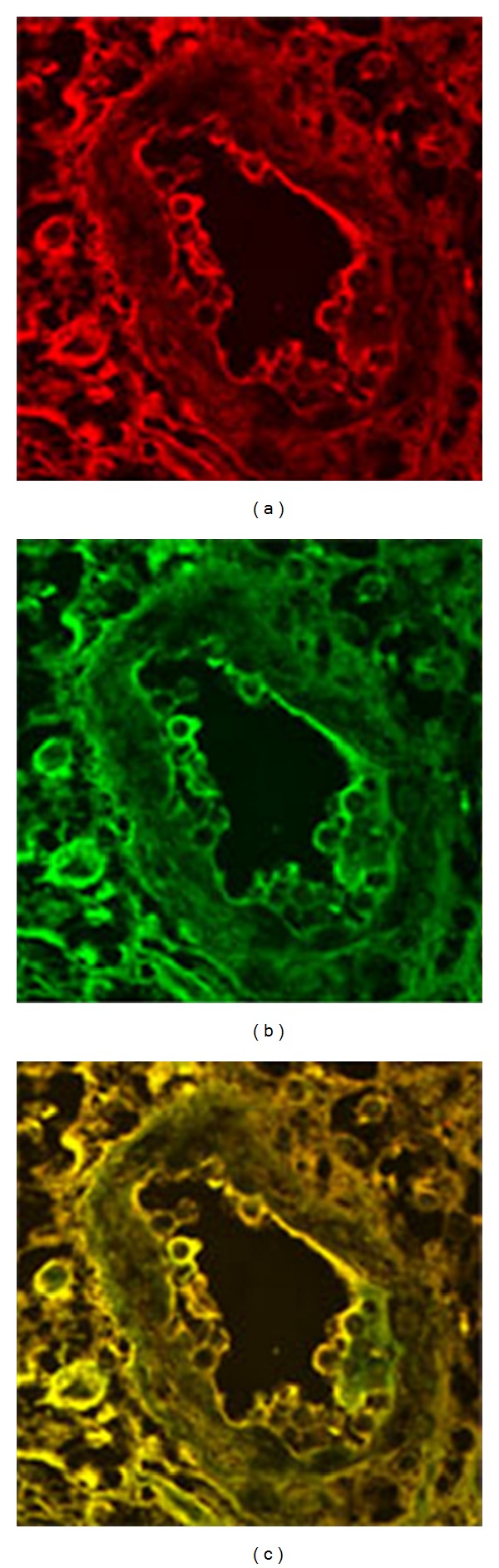
Hemangioma observed by confocal microscopy: (a) red immunofluorescence shows the overexpression and phosphorylation of the PDGF*β* receptor, (b) green immunofluorescence detects the expression of the E5 oncoprotein, and (c) colocalization of the receptor and the oncoprotein documented by yellow immunofluorescence of the merged image.

**Figure 7:**
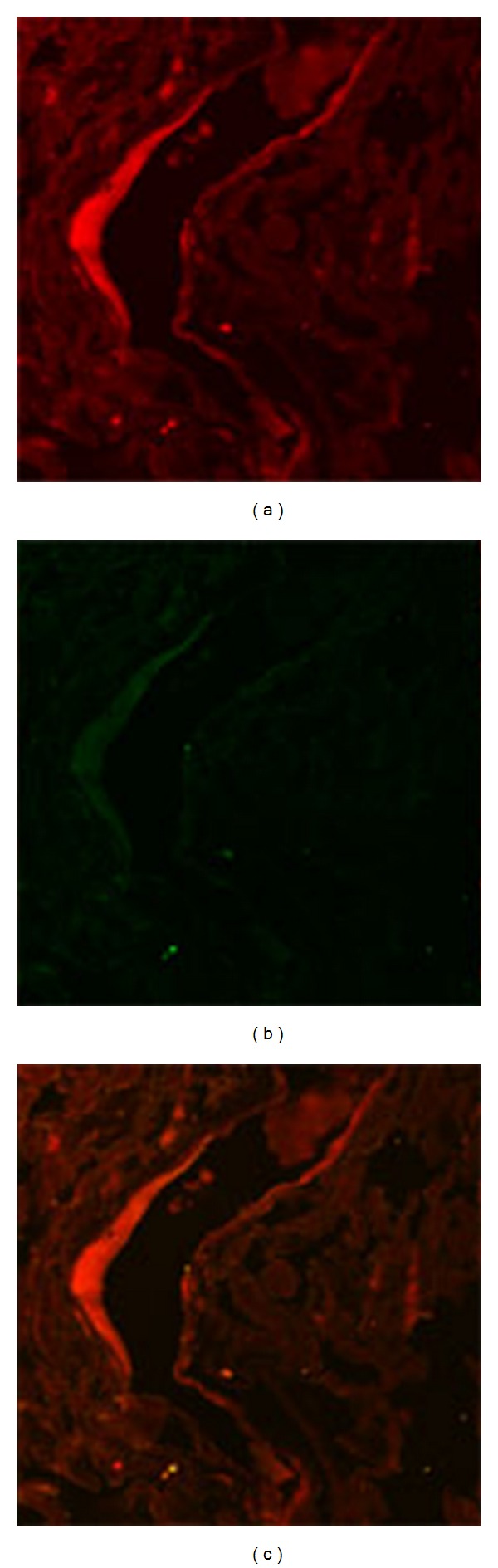
Normal vessel observed by confocal microscopy: (a) red immunofluorescence shows the expression and phosphorylation of the constitutive PDGF*β* receptor, (b) green immunofluorescence shows no E5 oncoprotein, and (c) immunofluorescence of the merged image failed to show any colocalization of E5 oncoprotein with PDGF*β* receptor.

 In forty vascular tumors, the colocalization of E5 oncoprotein with the overexpressed and phosphorylated PDGF*β*R was clearly revealed by the yellow fluorescence of the merged image by laser-scanning confocal microscopy (LSCM) in endothelial cells (Figures 6(a), 6(b), and 6(c)). LSCM failed to detect this complex in normal endothelial cells (Figures 7(a), 7(b), and 7(c)). These findings show that BPV-2 virus, the DNA of which was already documented in tumor samples from soft tissue tumors collected by the laser microdissection method as previously reported [37], produces an active infection in mesenchymal cells of the urinary bladder and also plays an important role in the development of cancers of the soft tissue of the bovine urinary bladder.

## 4. Discussion 

 In human medicine mesenchymal tumors of the urinary bladder are uncommon neoplasms [35]. Soft tissue tumors and tumor-like lesions of the bladder are generally described in isolated case reports and short series [30, 35] and are by far less common than epithelial lesions.

 Mesenchymal tumors are quite common in cattle harboring papillomavirus infection and grazing on lands rich in bracken fern, which shows that E5 protein, the major oncoprotein of BPV-2 and ptaquiloside and the major oncogenic factor of bracken fern, may play a synergistic role also in mesenchymal cancerogenesis of the urinary bladder.

 It has been suggested that hemangiomas are congenital lesions arising from embryonic angioblastic stem cell [32, 39]. Some authors prefer to include arteriovenous malformation (AVM) of the urinary bladder in the spectrum of hemangiomas [35]. Furthermore, it is believed that hemangiomas diagnosed in adults are malformations detected later in life [30]. 

 We never observed hemangiomas of the urinary bladder alone. These lesions were seen in association with other mesenchymal tumors but, mostly, with urothelial tumors in adult cattle. In addition, we detected, for the first time, that E5 oncoprotein binds to the activated (phosphorylated) form of PDGF*β*R that appears to be overexpressed in the endothelial cells of some hemangiomas. These findings allow us to suggest that bladder hemangiomas in cattle are not congenital malformations. We believe that the partnership between E5 oncoprotein and PDGF*β*R plays a crucial role also in mesenchymal bladder cancerogenesis in cattle. These observations confirm that BPV-2 is able to infect mesenchymal cells and corroborate our previous reports in which we showed that E5 oncoprotein interaction with PDGF*β*R is responsible for urothelial cell transformation [20]. It is worthwhile remembering that PDGF-B/PDGF*β*R is constitutively expressed in endothelial as well as in vascular smooth muscle cells (vSMCs) and pericytes, thus appearing to play a central role in vascular development [40]. Abnormal expression of PDGF-B and PDGF*β*R is associated with a severe vascular pathology [40]. Furthermore, it has been shown that CD4(+) and CD8(+) lymphocytes represent the most important reservoir of active BPV-2 in the blood of cattle as E5 oncoprotein has been detected in defined subsets of the peripheral blood mononuclear cells (PBMCs) [41]. Very likely, the high incidence of vascular tumors found in cattle suffering from chronic enzootic hematuria must be attributable to the transformation potential of E5 oncoprotein via the activation of the PDGF*β*R, which makes cattle unique among large animals.

 There are fewer than 30 cases of primary angiosarcomas of the urinary bladder in man [35]. They are considered to be aggressive, high-grade tumors. Prognosis is poorer than angiosarcomas in other sites. Regional lymph nodes are typically spared but local recurrence with distant metastasis is the rule [42]. These patients tend to have rapidly progressing disease and poor long-term survival. 

 In cattle angiosarcomas of the urinary bladder are the most frequent malignant mesenchymal tumors. Most of them are epithelioid angiosarcomas, the cells of which share many morphological features with human angiosarcoma cells. Despite an aggressive appearance histologically, bovine angiosarcoma has a very low potential of metastasis. Out of 23 angiosarcoma in this series, only one showed metastasis to distant organs. 

 In papillomavirus-associated bladder tumors, severe diffuse inflammation is invariably present in the stroma of both urothelial and mesenchymal tumors [4, 43]. It is well known that papillomavirus L1 protein plays a central role both in infection and immunogenicity [44]. It has just been shown that L1 protein is surprisingly expressed in high-grade bladder cancer of ruminants [45]. L1 protein expression may contribute to chronic inflammation of tumor stroma known to have an important role in the progression and metastasis of tumors. It has been suggested that immune cells play an emerging role in controlling tumor metastasis [46, 47]. 

 Therefore, it is reasonable to believe that the L1-mediated severe inflammation of tumor stroma might play a role in controlling the metastatic potential of bladder tumors. Better understanding of the relationship, if any, between biological behavior of urothelial and mesenchymal tumors of ruminants and their stromal inflammation will be an important area of work in the future. 

 Finally, we report the first cases of IPEH of the urinary bladder in animals. Our work seems to strengthen the hypothesis that IPEH is not a true tumor but rather an exuberant organization and recanalization of a thrombus. 

 As spontaneous bladder pathology is quite common in cattle grazing on fern-infested pastures, we suggest that this species may serve as a spontaneous biological model that should prove rich ground for future research in papillomavirus biology and comparative bladder oncogenesis.

## Figures and Tables

**Table 1:** Histopathological types and number of mesenchymal tumors and tumor-like lesions in the bovine urinary bladder.

Details of tumors	No.	Percentage (%)
Tumors of vascular origin	*204 *	~*81% *
Hemangioma	143	57%
Hemangioendothelioma	2	0.8%
Angiosarcoma	57	23%
Lymphangioma	2	0.8%
Hemangiopericytoma	9	3.6%
Glomus tumor	1	0.4%
Tumors of muscle origin	*16 *	~*6% *
Leiomyoma	5	2%
Leiomyosarcoma	8	3.2%
Rhabdomyosarcoma	3	1.2
Miscellaneous mesenchymal neoplastic lesions	*23 *	*9% *
Fibroma and stromal fibrous reaction	5	2%
Myxoma and stromal myxomatous reaction	13	5.2%
Fibrosarcoma	2	0.8%
Malignant lymphoma	3	*~1% *

Total	253	100%
